# Adequate serum 25-hydroxy-vitamin D levels are correlated with low anti-PF4 levels in mild COVID-19 Patients: An observational study

**DOI:** 10.1097/MD.0000000000039252

**Published:** 2024-09-13

**Authors:** Andhika Rachman, Anggraini Iriani, Attaufiq Irawan, Samuel Juanputra, Rachelle Betsy

**Affiliations:** a Division of Hematology and Oncology, Department of Internal Medicine, Dr Cipto Mangunkusumo National Referral Hospital, Faculty of Medicine, Universitas Indonesia, Jakarta, Indonesia; b Department of Clinical Pathology, YARSI University, Jakarta, Indonesia; c Department of Internal Medicine, Dr Cipto Mangunkusumo General Hospital – Faculty of Medicine Universitas Indonesia, Jakarta, Indonesia.

**Keywords:** anti-PF4, COVID-19, P-selectin, serum 25(OH)D, vitamin D

## Abstract

The worldwide spread of coronavirus disease 2019 (COVID-19) has resulted in an unparalleled health emergency of global proportions. Around 31% of individuals with COVID-19 experience thrombosis associated with hypercoagulation. COVID-19 patients have shown an increase in platelet activation, but the mechanism has not been fully understood yet. One theory suggests that this could be related to the heparin-induced thrombocytopenia phenomenon, where platelet activation involves anti-PF4 antibodies that are associated with thrombosis. Vitamin D has been established to exert an influence on immunological responses and inflammation. The aim of this study is to analyze the correlation between serum 25-hydroxy-cholecalciferol [25(OH)D] levels and anti-PF4 antibodies among COVID-19 patients. A cross-sectional study was conducted among 160 COVID-19 patients at Cipto Mangunkusumo General Hospital and Wisma Atlit Hospital Jakarta from October 2021 to January 2022. The mean serum 25(OH)D level was 15.1 ng/mL. A significant negative correlation was found between serum 25(OH)D and anti-PF4 levels in mild COVID-19 patients (*P* = .035; *R* = −0.236). Remarkably, P-selectin levels were significantly higher in the moderate COVID-19 group compared to the severe group (*P* = .031). Serum 25(OH)D level had a significant negative correlation with anti-PF4 level in mild COVID-19 patients. Thus, it is highly recommended to ensure that serum 25(OH)D levels are maintained above 30 ng/mL. Remarkably, the P-selectin level was significantly higher in the moderate COVID-19 group compared to the severe group.

## 1. Introduction

The global spread of coronavirus disease 2019 (COVID-19) has led to an unparalleled health crisis on a global scale.^[1]^ Declared a pandemic by the WHO in March 2020, this virus has affected millions globally, including in Indonesia, which reported its first case in March 2020. Although the mortality rate in Indonesia is not among the highest, the implications of COVID-19 are profound, especially regarding complications such as thrombosis.^[2–4]^

In the context of thrombosis, 31% of COVID-19 patients experience this condition, with the risk increasing in parallel with the disease severity. Thrombosis in COVID-19 has been associated with hypercoagulability, as evidenced by elevated d-dimer levels or conditions like SIC (sepsis-induced coagulopathy) and DIC (disseminated intravascular coagulation). Recent studies indicate a significant increase in the risk of thrombosis among COVID-19 patients. A systematic review assessed the performance of viscoelastic methods (VEMs) in COVID-19 patients. The studies reported that these patients displayed hypercoagulability, characterized by increased maximum amplitude in thromboelastography (TEG) and high maximum clot firmness in thromboelastometry (TEM), indicating strong clot formation. Additionally, fibrinolysis shutdown was observed, with reduced or absent fibrinolytic activity (LY30). Despite the use of thromboprophylaxis, the incidence of thrombotic events remained high, with venous thromboembolism (VTE) reported in up to 49% of patients.^[5]^ Consequently, anticoagulants are recommended for moderate-to-severely affected COVID-19 patients.^[2–4]^ However, the role and presence of platelets in thrombosis in COVID-19 patients still require a deeper understanding.^[2,6]^

Several studies have indicated increased platelet activation in COVID-19 patients, yet the mechanism remains not fully understood. One hypothesis suggests a phenomenon akin to heparin-induced thrombocytopenia, where platelet activation occurs through antibodies targeting platelet‐factor 4 (anti-PF4)/ heparin associated with thrombosis.^[6–8]^ Generated by platelets, PF4 has the ability to stimulate the development of antibodies that can attach to PF4. The interaction between PF4 and the antibody can initiate platelet actication, hence increasing the risk of thrombosis.^[9]^ Therefore, specific antibodies in COVID-19, especially anti-PF4 might affect platelet activation and resultant thrombosis.^[7,8]^

On the other hand, vitamin D has been associated with many health functions, including immune response and inflammation. Recent studies demonstrated that vitamin D plays a preventive role in thrombotic disorders. The possible mechanism responsible for the reported effects of vitamin D involves the potential regulation of inflammatory responses by increasing the expression of IL-10 receptors and decreasing the activation of NF-κB. Vitamin D can decrease oxidative stress in endothelial cells, leading to a decrease in the production of blood clots.^[10–13]^ Understanding the correlation between vitamin D status and specific antibodies like anti-PF4 is crucial in the context of COVID-19.^[10,11]^ While several studies have examined the role of vitamin D and anti-PF4 in COVID-19, none have specifically analyzed the relationship between the 2. Based on current evidence, we hypothesize that anti-PF4 may potentially have an impact on the level of serum 25(OH)D. This research aims to analyze the correlation between serum 25(OH)D and anti-PF4 antibodies in COVID-19 patients.

## 2. Methods

This cross-sectional study was conducted at Dr Cipto Mangunkusumo General Hospital (RSCM) and Wisma Atlit Jakarta Hospital between October 2021 and January 2022. The inclusion criteria consisted of individuals of both genders, aged 18 years or older, who were diagnosed with COVID-19 using the PCR method. This study did not limit inclusion to only first-onset cases. The exclusion criteria included pregnant women, individuals with trauma or active bleeding, those using antiplatelet medication, and those with a history of hypercoagulable syndrome. We did not exclude vaccinated patients from our analysis.

At the time of hospital admission, serum samples were taken from individuals with COVID-19. The kits for measuring P-selectin and anti-PF4 levels were produced by Cloud-clone Corp., USA, and the kit for measuring serum 25-hydroxy-vitamin D levels was produced by Roche Diagnostics. Soluble P-selectin and anti-PF4 levels were measured using the enzyme-linked immunosorbent assay (ELISA) method in a microplate reader, as per the instructions provided by the kit manufacturer (Cloud-clone Corp., USA). Assay range of the soluble P-selectin kit was 1 to 140 ng/mL and minimal detection limit was approximately 1.0 ng/mL. The intraassay and interassay coefficients of variation (CV) for the assay were < 5% and < 8%, respectively. The PF4 kit had an assay range of 1 to 100ng/mL and minimal detection limit of approximately 1.0 ng/mL. The intraassay and interassay CV for the assay were < 8% and < 10%, respectively. The concentration of serum 25-hydroxyvitamin D [25(OH)D] is utilized to determine vitamin D status. Measurements are conducted using a competitive electrochemiluminescence protein binding assay with Cobas e411 from Roche Diagnostics. Assay range of the Cobas e411 was 3 to 100 ng/mL and minimal detection limit was 3 ng/mL. The intraassay and interassay CV for the assay were <7% and <10%, respectively. Comorbidities among the study participants were identified and categorized as follows: chronic heart disease (CAD/CHF), type 2 diabetes mellitus (T2DM), chronic kidney disease (CKD), cerebrovascular disease, autoimmune conditions, liver diseases, hypertension, HIV, chronic obstructive pulmonary disease (COPD), and cancer.

### 2.1. Statistical analysis

The statistical analyses in this study were performed using the Statistical Package for the Social Sciences (SPSS, Chicago, IL) version 27 for Windows. All graphs or plots were created using GraphPad Prism v9.5.1 software for the Windows operating system. The bivariate analysis utilized the 1-way ANOVA test. Pearson correlation tests determined the correlation between serum 25(OH)D and anti-PF4 levels. Linear regression tests provided coefficient estimates.

### 2.2. Ethics approval

The study received ethical approval from the Ethics Committee of the Faculty of Medicine, Universitas Indonesia, with clearance number KET-1016/UN2.F1/ETIK/PPM.00.02/2022. The Declaration of Helsinki ethical guidelines were strictly followed throughout the investigation.

## 3. Results

In this study, a total of 160 individuals were included. The mean age of participants was 42.8 ± 16.2 years old. Of the subjects, 63 (39.4%) were male, and 97 (60.6%) were female. The average BMI was 22.8 ± 3.8 kg/m^2^. The mean 25(OH)D (25-hydroxy-cholecalciferol) level was 15.1 ± 8.1 ng/mL.

Various types of comorbidities identified in participants included CAD (coronary artery disease)/CHF (congestive heart failure) (8.2%), autoimmune conditions (3.8%), type 2 DM (diabetes mellitus) (33.3%), liver diseases (3.1%), hypertension (27.5%), CKD (8.2%), HIV (human immunodeficiency virus) (1.3%), COPD (1.3%), cerebrovascular diseases (3.1%), and cancer (7.5%). The mean hemoglobin value in participants was 13.6 ± 2.5 g/dL. The mean leukocyte count was 8827.2 ± 7554.7 cells/uL. The average platelet count was 291,800 ± 95.951.6 cells/uL. The mean d-dimer level was 1.610.9 ± 3146.8 ng/mL. The average creatinine level was 0.9623 ± 0.7 mg/dL.

Regarding COVID-19 status, the majority of participants (86.9%) had mild to moderate status, while 21 individuals (13.1%) had severe status. The comprehensive baseline characteristics of the participants can be seen in Table 1.

**Table 1:** Baseline characteristics of the participants.

Variables	N = 160
Age, mean ± SD	42.8 ± 16.2
BMI, mean ± SD	22.8 ± 3.8
Gender, N (%)	
Male	63 (39.4%)
Female	97 (60.6%)
Number of comorbidities, N (%)	
None	67 (41.9%)
1	28 (17.5%)
2	48 (30%)
3	15 (9.4%)
4	2 (1.3%)
Types of comorbidities, N (%)	
CAD	13 (8.2%)
Autoimmune	6 (3.8%)
T2DM	53 (33.3%)
Liver	5 (3.1%)
Hypertension	44 (27.5%)
CKD	13 (8.2%)
HIV	2 (1.3%)
COPD	2 (1.3%)
CVD	5 (3.1%)
Cancer	12 (7.5%)
COVID-19 vaccination status, N (%)	
None	44 (27.5%)
Once	3 (1.9%)
Twice	112 (70%)
Thrice	1 (0.6%)
Hemoglobin, mean ± SD	13.6 ± 2.5
Leukocytes, mean ± SD	8827.2 ± 7544.7
Platelets, mean ± SD	291,800 ± 95,951.6
D-dimer, mean ± SD	1610.9 ± 3146.8
P-selectin, mean ± SD	50.62 ± 48.27
Anti-PF4, mean ± SD	32.96 ± 34.62
Creatinine, mean ± SD	0.9623 ± 0.7
Serum 25(OH)D, mean ± SD, in ng/mL	15.1 ± 8.1
COVID-19 Status, N(%)	
Mild–moderate	139 (86.9%)
Severe	21 (13.1%)

Abbreviations: BMI, body mass index; CAD, coronary artery disease; CVD, cerebrovascular disease; CKD, chronic kidney disease; COPD, chronic obstructive pulmonary disease; HIV, human immunodeficiency virus; SD, standard deviation; T2DM, type 2 diabetes mellitus.

In the basic characteristics of the research subjects based on vitamin D status, the mean ages for the deficient, insufficient, and sufficient groups were 43.6 ± 16.1, 38.9 ± 17.4, and 35.9 ± 16.4 years, respectively. The proportion of males and females who were deficient was 77.7% and 85.1%, respectively, while in the insufficient group, the proportions were 17.5% and 9.6%. The proportion of males in the sufficient group was 4.8%, whereas females were 5.3%, with *P* = .348. Most subjects with mild to moderate and severe COVID-19 statuses tended to be deficient at 80.9% and 90.5%, respectively, while the proportion of those insufficient was 13.2% and 9.5% with *P* = .441. The basic characteristics of participants based on vitamin D status can be viewed in detail in Table 2.

**Table 2:** The basic characteristics of participants based on vitamin D status.

Variables	Status vitamin D			*P* value
	Deficient (≤20ng/mL)	Insufficient (21–29 ng/mL)	Sufficient (≥30ng/mL)	
Age, mean ± SD	43.6 ± 16.1	38.9 ± 17.4	35.9 ± 16.4	.232
BMI, mean ± SD	22.7 ± 3.8	23.6 ± 3.2	23.1 ± 4.1	.548
Gender, N (%)				
Male	49 (77.7%)	11 (17.5%)	49 (4.8%)	.348
Female	80 (85.1%)	9 (9.6%)	5 (5.3%)	
Number of comorbidities, N (%)				
None	50 (74.6%)	9 (13.4%)	80 (11.9%)	.137
1	24 (85.7%)	4 (14.3%)	0 (0%)	
2	40 (87%)	6 (13%)	0 (0%)	
3	13 (92.9%)	1 (7.1%)		
4	2 (100%)	0 (0%)	0 (0%)	
Type of comorbidities, N (%)				
CAD	11 (84.6%)	2 (15.4%)	0 (0%)	.667
Autoimmune	4 (66.7%)	2 (33.3%)	0 (0%)	.280
T2DM	44 (84.6%)	8 (15.4%)	0 (0%)	.107
Liver	5 (100%)	0 (0%)	0 (0%)	.568
Hypertension	37 (88.1%)	5 (11.9%)	0 (0%)	.198
CKD	12 (100%)	0 (0%)	0 (0%)	.241
HIV	1 (100%)	0 (0%)	0 (0%)	.896
COPD	2 (100%)	0 (0%)	0 (0%)	.801
CVD	4 (80%)	1 (20%)	0 (0%)	.789
Cancer	12 (100%)	0 (0%)	0 (0%)	.241
COVID-19 vaccination status, N (%)				
None	39 (92.8%)	3 (7.2%)	0 (0%)	.409
Once	2 (100%)	0 (0%)	0 (0%)	
Twice	87 (77.7%)	17 (15.2%)	8 (7.1%)	
Thrice	1 (100%)	0 (0%)	0 (0%)	
Hemoglobin, mean ± SD	13.6 ± 2.4	13.6 ± 2.9	14.7 ± 1.9	.460
Leukocytes, mean ± SD	9166.3 ± 8387.9	7320.5 ± 1153.3	7673.8 ± 2052.6	.550
Platelets, mean ± SD	298,142.8 ± 97,092.5	261,000 ± 83,188.4	278,375 ± 98,015.9	.254
D-dimer, mean ± SD	1864.6 ± 3508.7	998.3 ± 977.2	407.6 ± 277.2	.489
Creatinine, mean ± SD	0.9 ± 0.7	1.04 ± 0.6	0.7 ± 0.2	.619
Covid-19 status, N (%)				
Mild-moderate	110 (80.9%)	18 (13.2%)	8 (5.9%)	.441
Severe	19 (90.5%)	2 (9.5%)		

Analyzed using 1-way ANOVA test.

Abbreviations: BMI, body mass index; CAD, coronary artery disease; CVD, cerebrovascular disease; CKD, chronic kidney disease; COPD, chronic obstructive pulmonary disease; HIV, human immunodeficiency virus; SD, standard deviation; T2DM, type 2 diabetes mellitus.

**Figure 1.**
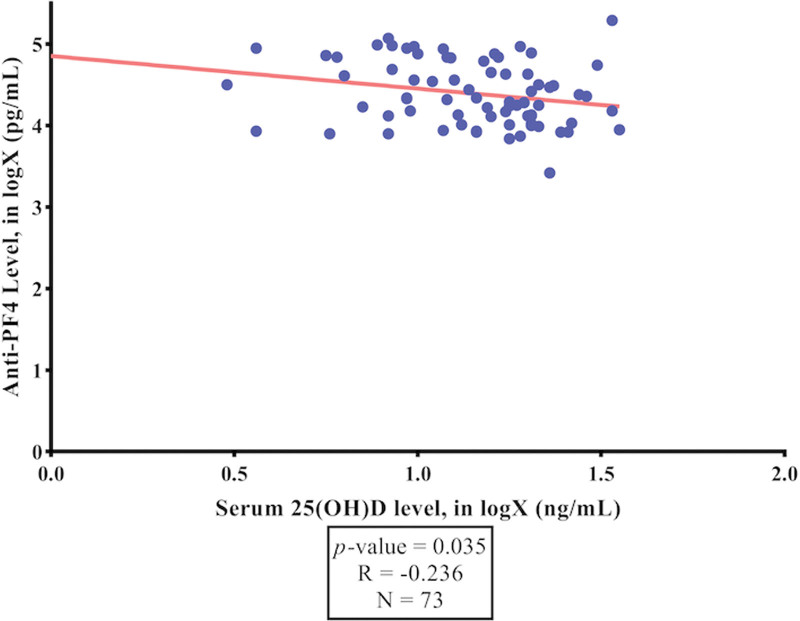
The correlation between serum 25(OH)D levels and anti-PF4 level among mild COVID-19 Patients. Analyzed using Pearson correlation test. Abbreviation: 25(OH)D, 25-hydroxy-vitamin D.

As can be seen in Figure 1, there was a significant negative correlation between the level of serum 25(OH)D and the anti-PF4 level in mild COVID-19 patients (*P* = .045; R = −0.236). Based on the results of the simple linear regression test, the formula for anti-PF4 (antibodies targeting platelet‐factor 4), in logX (pg/mL) = 4.85–0.4 log(serum 25(OH)D [ng/mL]), was obtained.

Furthermore, the mean level of P-selectin in the mild COVID-19 group is 55.46 ± 42.21 ng/mL, in the moderate COVID-19 group is 69.02 ± 57.06 ng/mL, and in the severe COVID-19 group is 37.03 ± 34.60 ng/mL (Table 3).

**Table 3:** Levels of P-selectin and anti-PF4 variables based on COVID-19 status.

Variables	COVID-19 status			*P* value
	Mild (N = 80)	Moderate (N = 59)	Severe (N = 21)	
P-Selectin, mean ± SD, in ng/mL	55.46 ± 42.21	69.02 ± 57.06	37.03 ± 34.60	**.037**
Anti-PF4, mean ± SD, in ng/mL	36.94 ± 34.61	31.39 ± 36.60	25.63 ± 28.47	.120

Analyzed using 1-way ANOVA test.

Abbreviations: SD = standard deviation.

Bold indicates a statistically significant.

**Figure 2.**
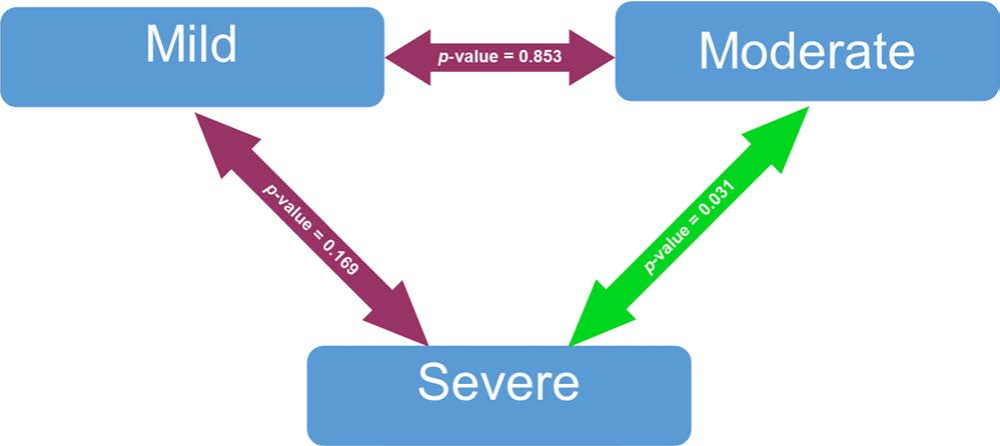
The Bonferroni post hoc test for P-selectin based on COVID-19 severity. Analyzed using Bonferroni post hoc test. Significant at *P*-value < 0.05. The green double-arrow denotes statistically significant difference. The red double-arrow denotes nonstatistically significant difference.

Interestingly, the P-selectin level was significantly higher in the moderate COVID-19 group compared to the severe group (*P* = .031) (Fig. 2; Table 3).

## 4. Discussion

To the best of our knowledge, this is the first study to analyze the relationship between serum 25(OH)D levels and anti-PF4 in COVID-19 patients. We found there was a significant negative correlation between anti-PF4 level and serum 25(OH)D level in mild COVID-19 patients (Fig. 1). This result indicates that as the concentration of vitamin D increases, the concentration of anti-PF4 tends to decrease, and conversely. Anti-PF4 has been established as an antibody recognizing PF4. PF4 is a cytokine released by platelets capable of inducing antibody production that can bind with PF4. The formation of complexes between PF4 and the antibody can trigger platelet activation, enhancing thrombosis risk.^[9]^ Vaccine-induced thrombotic thrombocytopenia associated with adenoviral COVID-19 vaccines results in anti-PF4 antibodies, triggering thrombosis in patients.^[14]^ On the other hand, a cross-sectional study by Tao et al revealed that vitamin D has a protective function in thrombolytic diseases. The potential mechanism underlying the observed effects of vitamin D involves the potential modulation of inflammatory responses by upregulating IL-10 receptor expression and downregulating NF-κB activation. Vitamin D may reduce oxidative stress in endothelial cells, thereby reducing blood clot formation. Further, vitamin D and its analogues were reported to alter coagulation pathways, either directly or indirectly, suggesting they may have antithrombotic characteristics. Another pilot randomized clinical trial by Hejazi et al, demonstrated that vitamin D supplementation improved the anticoagulation effectiveness of warfarin in patients with deep vein thrombosis and pulmonary embolism.^[12,13]^ According to the findings from a cellular study by Ohsawa et al, vitamin D exhibited potential inhibitory effects on coagulation in monocytic cells. This was achieved through the downregulation of tissue factor and the upregulation of thrombomodulin (TM), as well as the reduction of the impact caused by tumor necrosis factor and oxidized low-density lipoprotein. These actions collectively contributed to the manifestation of antithrombotic activity.^[13,15]^ Additional mechanisms of anticoagulation have been identified in previous studies. These mechanisms include the modulation of plasminogen activator inhibitor-1 (PAI-1) and the expression of thrombospondin-1 in smooth cells. Furthermore, the downregulation and modulation of various coagulation indicators, such as highly sensitive C-reactive protein, tissue factor pathway inhibitors, and TNF-α, have also been observed. These findings contribute to a better understanding of the complex processes involved in anticoagulation.^[13,16]^ Vitamin D has been shown in several studies to reduce inflammation by lowering the levels of cytokines that cause inflammation. These include IL-6, IL-8, IL-12, IL-17, NF-kB, TNFα, gamma interferon (IFN-y), and IL-2. Hence, this mechanism contributes to reducing oxidative stress and preventing cellular damage in individuals affected by COVID-19.^[16]^ Our findings have strengthened previous theories that vitamin D deficiency might affect endothelial function and elevate an inflammatory response, promoting coagulation.^[17]^ Therefore, we highly recommend that COVID-19 patients maintain serum 25(OH)D levels above 30 ng/mL, as it will be beneficial to reduce inflammation and improve the outcome of the disease.^[10,11]^

Furthermore, this study also found that P-selectin was significantly higher in the moderate COVID-19 group compared to the severe group (Fig. 2; Table 3). Our finding was supported by a large cohort study by Fenyves et al that demonstrated P-selectin as an early indicator of thromboembolism in COVID-19 patients.^[18]^ P-selectin is a crucial molecule in the process of thrombo-inflammation, playing a significant role in the activation and functioning of platelets. Extensive research has shown that it plays a pivotal role in primary hemostasis through its ability to regulate various processes. These include platelet–leukocyte interactions, the recruitment of fibrin and tissue factor into platelet aggregates, and the formation of thrombus. The soluble form of P-selectin is released when platelets and endothelial cells are activated. Previous studies have suggested that measuring soluble P-selectin levels could serve as a dependable indicator of platelet activation in vivo.^[19]^ A cohort study by Chao et al revealed that platelet activation is an initial response observed in mild or moderate COVID-19, and it is not primarily linked to severe manifestations of the disease.^[19,20]^ Platelets play a crucial role in both coagulation and immune responses, making them valuable biomarkers for identifying potential therapeutic targets related to inflammation or coagulation dysfunction in COVID-19. The observed platelet activation phenotype in COVID-19 highlights the ability of platelets to mount a response to viral infections. Recent research suggests that platelets may have the ability to uptake the SARS-CoV-2 virus. A study has shown that hyperactivated platelets derived from individuals with COVID-19 express SARS-CoV-2 RNA.^[19–21]^ The potential value of monitoring platelet counts in COVID-19 has been reported by Yang et al.^[19,20,22,23]^ However, it is proposed that monitoring platelet activation may potentially enhanced predictive value, as it may occur prior to any changes in platelet counts. Thus, antiplatelet therapy has been proposed as a potential treatment option for COVID-19. The potential benefits of antiplatelet therapy in terms of protection and therapy during the progression of COVID-19 have been suggested, leading to the need for additional research in this area.^[19,20,24]^

The strength of our study is the fact that it is the first study to provide evidence of a significant correlation between serum 25(OH)D levels and anti-PF4 in mild COVID-19 patients. Nevertheless, there is a limitation that must be considered when interpreting our findings. The present investigation utilizes a cross-sectional research design. Therefore, more interventional trials, such as a randomized control study, are suggested to validate our findings.

## 5. Conclusion

We found that serum 25(OH)D level had a significant negative correlation with anti-PF4 level in mild COVID-19 patients. The P-selectin level was significantly higher in the moderate COVID-19 group compared to the severe group. It is important to emphasize that this is a pilot study with a limited sample size, aimed at exploring initial trends and associations. Hence, further studies are needed to evaluate the potential benefits of antiplatelet therapy for the management of COVID-19, as it exhibits promising potential for reducing the morbidity and mortality of COVID-19.

## Acknowledgments

The authors would like to thank the professional staff of Faculty of Medicine, Universitas Indonesia for their cooperation in this study.

## Author contributions

**Conceptualization:** Andhika Rachman, Anggraini Iriani, Attaufiq Irawan, Samuel Juanputra, Rachelle Betsy.

**Data curation:** Andhika Rachman, Anggraini Iriani, Attaufiq Irawan, Samuel Juanputra, Rachelle Betsy.

**Formal analysis:** Andhika Rachman, Anggraini Iriani, Attaufiq Irawan, Samuel Juanputra, Rachelle Betsy.

**Funding acquisition:** Andhika Rachman, Anggraini Iriani, Attaufiq Irawan, Samuel Juanputra, Rachelle Betsy.

**Investigation:** Andhika Rachman, Anggraini Iriani, Attaufiq Irawan, Samuel Juanputra, Rachelle Betsy.

**Methodology:** Andhika Rachman, Anggraini Iriani, Attaufiq Irawan, Samuel Juanputra, Rachelle Betsy.

**Project administration:** Andhika Rachman, Anggraini Iriani, Attaufiq Irawan, Samuel Juanputra, Rachelle Betsy.

**Resources:** Andhika Rachman, Anggraini Iriani, Attaufiq Irawan, Samuel Juanputra, Rachelle Betsy.

**Software:** Andhika Rachman, Anggraini Iriani, Attaufiq Irawan, Samuel Juanputra, Rachelle Betsy.

**Supervision:** Andhika Rachman, Anggraini Iriani, Attaufiq Irawan, Samuel Juanputra, Rachelle Betsy.

**Validation:** Andhika Rachman, Anggraini Iriani, Attaufiq Irawan, Samuel Juanputra, Rachelle Betsy.

**Visualization:** Andhika Rachman, Anggraini Iriani, Attaufiq Irawan, Samuel Juanputra, Rachelle Betsy.

**Writing – original draft:** Andhika Rachman, Anggraini Iriani, Attaufiq Irawan, Samuel Juanputra, Rachelle Betsy.

**Writing – review & editing:** Anggraini Iriani, Attaufiq Irawan, Samuel Juanputra, Rachelle Betsy.
